# Effects of resistance training on sleep quality and disorders among individuals diagnosed with cancer: A systematic review and meta‐analysis of randomized controlled trials

**DOI:** 10.1002/cam4.7179

**Published:** 2024-04-23

**Authors:** Dora Maric, Salvatore Ficarra, Luca Di Bartolo, Carlo Rossi, Zoi Asimakopoulou, Apostolos Vantarakis, Ana Carbonell‐Baeza, David Jiménez‐Pavón, Beatriz Gomes, Paula Tavares, Rebecca Baxter, Susanna Pusa, Petra Thaller, Sofia Papakonstantinou, Musa Kirkar, Francesca Glorioso, Marina Galioto, Ambra Gentile, Ewan Thomas, Antonino Bianco

**Affiliations:** ^1^ Sport and Exercise Sciences Research Unit, Department of Psychology, Educational Science and Human Movement University of Palermo Palermo Italy; ^2^ Division of Population Sciences, Department of Medical Oncology Dana‐Farber Cancer Institute Boston Massachusetts United States; ^3^ University of Patras Patras Greece; ^4^ MOVE‐IT Research Group, Department of Physical Education, Faculty of Education Sciences University of Cadiz Cadiz Spain; ^5^ Biomedical Research and Innovation Institute of Cadiz (INiBICA) Cadiz Spain; ^6^ CIBER of Frailty and Healthy Aging (CIBERFES) Madrid Spain; ^7^ Faculty of Sport Sciences and Physical Education University of Coimbra Coimbra Portugal; ^8^ Department of Nursing Umeå University Umeå Sweden; ^9^ Department of Health Consulting, Research and Science Outdoor Against Cancer München Germany; ^10^ Creative Thinking Development—CRE.THI.DEV Rafina Greece; ^11^ CEIPES ETS Palermo Italy; ^12^ Lega Italiana per la lotta Contro i Tumori (LILT Palermo) Palermo Italy

**Keywords:** exercise oncology, insomnia, physical activity, quality of life, tumor

## Abstract

**Background:**

Sleep disorders are often complained by cancer patients and can last years after the end of therapies, leading to different negative consequences. Non‐pharmacological strategies such as exercise interventions may be considered to counteract this phenomenon. The literature supports the beneficial effects of aerobic training (AT), while evidence on resistance training (RT) is scarce. Accordingly, our systematic review aims to investigate the potential novel effect of RT on sleep outcomes in cancer survivors.

**Methods:**

The literature search was conducted on MEDLINE (Pubmed), Web of Science, Scopus, and Cochrane Central Register of Controlled Trials databases, including only randomized controlled trials (RCTs). The screening procedure was conducted using the web‐based software COVIDENCE. Sleep outcomes assessed through self‐reported questionnaires or objective sleep measurements were extracted from RCTs recruiting cancer survivors of any age and gender, on or off treatment. The risk of bias (RoB) for each study was assessed using the Cochrane RoB 2 tool for RCTs. Meta‐analytic syntheses were performed on sleep quality and insomnia.

**Results:**

A total of 21 studies were included in the review. Considering the mean percentage differences of all studies combined, promising positive results were found after combined aerobic and resistance exercise program (COMB) for sleep quality (−19%) and sleep disturbance (−17.3%). The meta‐analysis results showed significant improvement for both sleep quality and insomnia (*d* = 0.28, SE: 0.11, *Z* = 2.51, *p* < 0.01, 95% CI: 0.07–0.49 and *d* = 0.43, SE: 0.20, *Z* = 2.18, *p* = 0.029, 95% CI: 0.07–0.49, respectively).

**Conclusion:**

RT interventions of 60 minutes per session, performed 2–3 times a week for 12 weeks, with exercise intensity ranging from 60% to 80% of one‐repetition maximum can be administered to cancer survivors, aiming to improve sleep outcomes.

## 1 INTRODUCTION

According to the Global Cancer Observatory, 19.3 million cancers were diagnosed and 10 million cancer deaths were estimated in 2020,^1^ leading cancer to be the second cause of death worldwide.^2^ Since 1991, cancer mortality has decreased due to treatment improvements and early diagnosis.^3^ Notwithstanding, cancer disease and therapies may cause different symptoms and conditions^4^ that can have a negative impact on patients' and survivors' quality of life (QoL).^5^

Sleep problems and disorders are experienced by 30%–50% (up to 95%) of cancer patients and can persist at every stage of the disease lasting up to 10 years during survivorship.^6^ This phenomenon could last longer due to its association with other cancer symptoms and side effects such as fatigue, depression, pain, and tiredness.^7^ Cognitive impairments are also experienced by cancer populations as sleep disorder consequences. The latter are faced more often by women diagnosed with breast cancer having insomnia when compared to those who are not living with this symptom.^8^ In order to treat sleep problems and disorders, pharmacological treatments are usually implemented.^6^ However, these approaches are not without side effects and may exacerbate other cancer symptoms, including headache or fatigue.^6^

Alternative strategies should be taken into account to face sleep disturbances. Non‐pharmacological strategies, such as exercise interventions administered as aerobic (AT) and resistance training (RT), can be valuable to cope with sleep disorders and limit drugs side effects, considering also its benefits on many other cancer‐related symptoms.^4^ A recent systematic review and meta‐analysis (2017) including both randomized and non‐randomized interventions investigated sleep outcomes after exercise in cancer patients.^9^ The review concluded that exercise did not significantly improve subjective or objective measures of sleep, although it mainly included AT interventions.^9^ However, the American College of Sports Medicine (ACSM) highlighted that recently published randomized controlled trials (RCTs) have shown the potential effects of AT^10^ and walking activities^11^, ^12^ on these outcomes. Therefore, moderate‐intensity AT (e.g., walking) for 30‐40 minutes, 3–4 times/week, to foster sleep improvements in cancer survivors was suggested.^4^ Conversely, evidence of sleep improvements in the cancer population after RT was insufficient, and this type of exercise was not suggested.^4^

Nevertheless, RT programs have been demonstrated to be beneficial for healthy individuals. The World Health Organization (WHO) guidelines suggest engaging in 150–300 minutes of moderate‐intensity or 75–150 minutes of vigorous‐intensity AT, along with two or more days a week of RT at moderate to vigorous intensity for all major muscle groups.^13^ These recommendations apply both for adolescents, adults, and older adults.^13^ RT can induce blood pressure reduction,^14^ muscle hypertrophy,^15^ increase bone mineral density,^16^ and, by secreting myokines (muscular cytokines) during muscle contractions, can stimulate different biochemical pathways.^15^ Furthermore, RT has also shown to be a valuable strategy for improving sleep outcomes in different populations.^17^ RT interventions have also been tested among cancer populations and are suggested to be administered alone or with AT 2–3 times a week to improve different symptoms and side effects,^4^ being also able to reduce overall cancer mortality.^18^ Moreover, exercise oncology research has significantly improved during the last few years, supporting the value of RT for cancer populations on other cancer symptoms.^19^, ^20^

Therefore, considering the potential benefits of RT on sleep outcomes and the development of the field of exercise oncology,^21^ we designed a systematic review and meta‐analysis to explore the potential novel effect of RT on sleep outcomes in cancer survivors, which are, according to the National Cancer Institute (NCI), those individuals from a cancer diagnosis until the end of life, living with cancer or cancer‐free.^22^ Establish if this exercise typology can influence this common disorder could be crucial for improving QoL among cancer populations in all stages of the disease.

## 2 METHODS

The existing review protocol has been registered in the PROSPERO database [CRD42023426762] and followed the updated Preferred Reporting Items for Systematic Reviews and Meta‐Analyses (PRISMA) guidelines.^23^

### 2.1 Search strategy

A relevant literature search was conducted starting in June 2023 on the following databases: MEDLINE (Pubmed), Scopus, Web of Science, and Cochrane Central Register of Controlled Trials. Only peer‐reviewed RCTs written in English without date limit were included. A preliminary search was conducted using “sleep AND (cancer OR tumor) AND training” to identify keywords through snowball sampling. Relevant keywords regarding the population included (individuals diagnosed with cancer) and the outcome (sleep) were evaluated and selected through discussion. The search string was created by combining the identified keywords using the Boolean operators “OR” and “AND”. The entire search strategy for each database is reported within the supplementary document. References obtained with the search were extracted from each database and were then imported into COVIDENCE,^24^ a web‐based collaboration software platform, to run the screening phases. The software initially removed duplicate records, which were then manually checked by one review author. Five review authors logged into COVIDENCE to carry out the title‐abstract and full‐text screening. Each study was assessed twice, and conflicts were resolved at each stage by the first author when needed. At the full‐text screening phase, unavailable full‐text authors were contacted. The impossibility of obtaining the manuscript led to its exclusion. References lists of selected relevant systematic and/or narrative reviews were also read to find additional literature to ensure a comprehensive screening process.

### 2.2 Inclusion and exclusion criteria

The following inclusion and exclusion criteria, listed following the participants, intervention, comparators, outcomes, and study design (PICOS**)** format, were applied during the entire screening process.

#### 2.2.1 Participants

Studies include cancer survivors^22^ of any age and sex. Individuals diagnosed with cancer were considered eligible (every stage and type of cancer), independently of existing treatment currently administered. The labels “on treatment” or “off treatment” were used to identify participants undergoing therapies with curative intent or at the end of treatments, respectively. The presence of co‐morbidities did not represent an exclusion criteria. Studies involving healthy individuals or animals were excluded.

#### 2.2.2 Intervention

Studies testing the effects of RT, defined as a training in which muscles exert a force against an external load,^25^ or a combination between AT and RT (COMB), were included to study the effects of RT or COMB protocols on sleep variables. Eligible types of RT were as follows: bodyweight training, resistance bands training, and conventional machine‐based/free weight RT. To isolate the effects of RT or COMB protocols, trials that included other approaches, such as behavioral management, acupuncture, physical therapy, and nutritional and psychological support sessions, were excluded.

#### 2.2.3 Comparators

Eligible comparator groups were: non‐exercising, stretching, and exercising control groups (CG) (e.g., AT only or other exercise types) for those off treatment and usual care groups during therapy administration. COMB or RT control groups were considered as intervention groups.

#### 2.2.4 Outcomes

Only sleep outcomes were considered. All sleep outcomes assessed using tests, such as patient‐reported questionnaires and/or accelerometer evaluations, were considered eligible. Various sleep outcomes, including global sleep quality, sleep disturbances, insomnia, daytime sleepiness, and sleep duration (hours/day), were selected and extracted.

#### 2.2.5 Study design

Only studies implementing a randomized design (RCTs) were included.

#### 2.2.6 Study record

All study characteristics were extracted and reported using an Excel spreadsheet to guarantee a comprehensive report of essential information. These included the following: sample size, groups, mean age, body mass index (BMI), sex, cancer site and stage, treatment phase, exercise interventions' characteristics, setting (supervised or home‐based), length (weeks), frequency (sessions/week), session duration (min), retention rate, and sleep assessment strategies. Studies were grouped according to the intervention type in two categories: RT alone and COMB interventions (RT+AT).

#### 2.2.7 Risk of bias assessment

Risk of bias (RoB) was assessed using the Cochrane RoB 2 tool for randomized trials^26^ by two review authors. Disagreements were discussed and solved to reach a final judgment for each of the included studies. This tool consists of five different domains, and each domain can be rated as low RoB, some concerns, and high RoB. The overall judgment of the trial was based on the judgment for each domain. If all the domains presented a low RoB judgment or at maximum one domain with some concerns, the overall study rating was low RoB. On the other hand, two to five domains with some concerns conducted to a “some concerns” overall rating. If at least one high RoB judgment was present, the trial was rated as high RoB.

#### 2.2.8 Data processing

Study characteristics were extracted. Numerical characteristics were summarized using median and interquartile ranges [IQR] (first and third). Baseline and post‐test data were extracted from each included study, and percentage differences were calculated for both intervention and exercise groups by two reviewers individually. Extracted and calculated data were cross‐compared between the two authors. Results are presented as mean ± standard deviation (SD) and percentage differences between post‐test and baseline data. Means of percentage differences were calculated for each type of outcome.

#### 2.2.9 Meta‐analytic synthesis

Two meta‐analytic syntheses were performed on sleep quality and sleep disturbance/insomnia through the metafor package of the R software (version 4.3.2). Mean, SD, and sample size were noted for each study. A total sample of *k* = 10 effects were collected concerning sleep disturbance/insomnia, while *k* = 6 studies were included about sleep quality. The meta‐analysis adopts the Hedges and Olkin approach,^27^ that refers to Cohen's *d* as a difference between the experimental condition mean and the control condition mean in standard units. Mean effect size was estimated referring to the random effect model. Heterogeneity of the studies was evaluated through Cochrane's *Q*, although moderation analysis was not performed due to the scarcity of studies about insomnia and sleep quality.

## 3 RESULTS

### 3.1 Study selection

**FIGURE 1:**
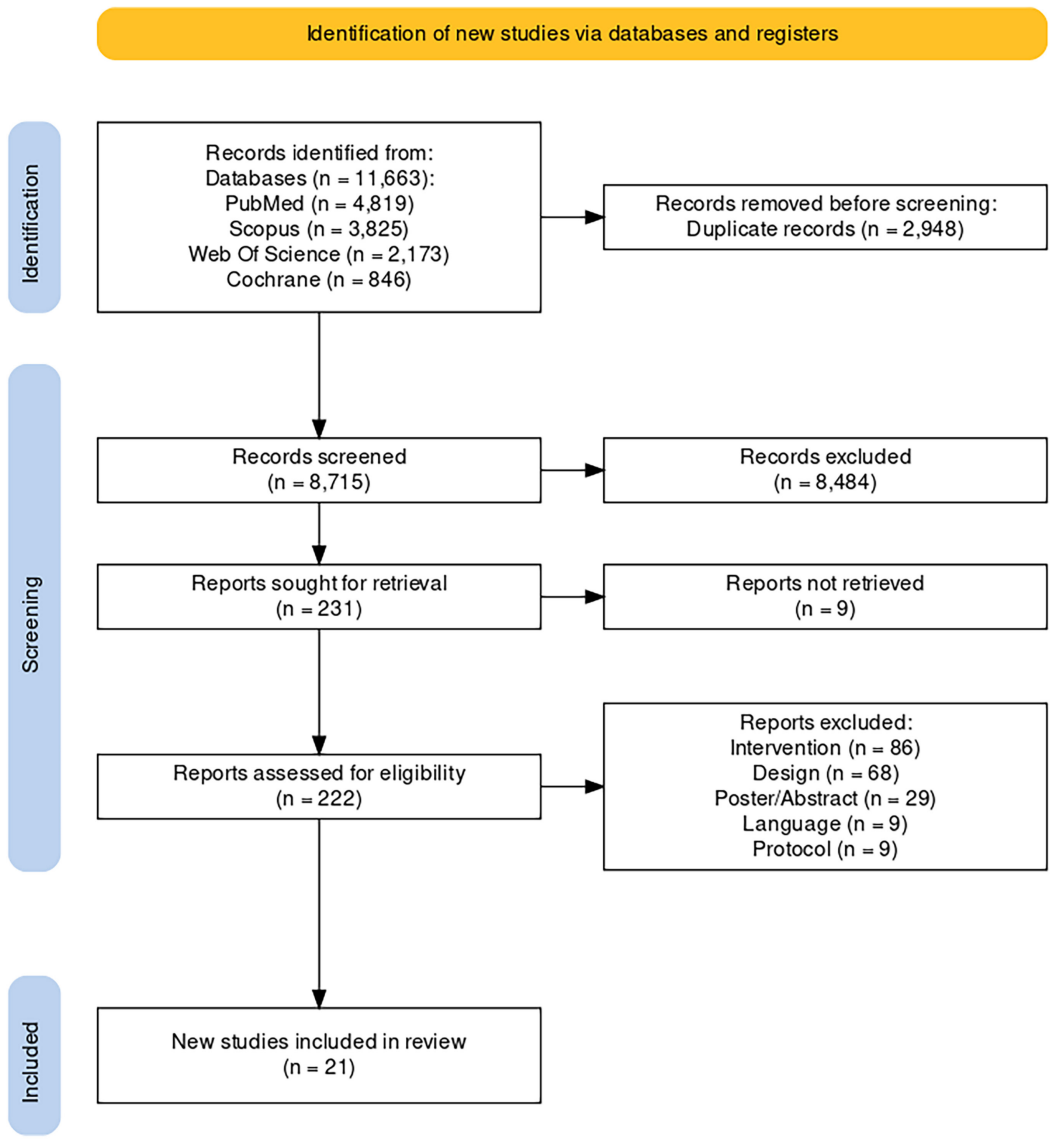
The PRISMA flow diagram of the screening process.

The screening procedure is presented in Figure 1 and generated using the PRISMA flow diagram app.^28^ Briefly, after the initial database search, 11,663 studies were identified, while 2948 duplicates were removed automatically. Removed duplicates were manually checked. After titles and abstract screening, 222 studies were assessed for eligibility, and 201 were excluded with reasons. A total of 21 studies were included in the review.

### 3.2 Study characteristics

Participants' characteristics and details regarding the RT interventions are reported in Tables 1 and 2, respectively. A total of 1.920 participants were included. Three studies (14.3%) included only male participants,^29^, ^30^, ^31^ 6 studies (28.6%) included solely female participants,^32^, ^33^, ^34^, ^35^, ^36^, ^37^ and 12 studies (57.1%) included participants of both genders.^38^, ^39^, ^40^, ^41^, ^42^, ^43^, ^44^, ^45^, ^46^, ^47^, ^48^, ^49^ Out of 21 RCTs, 19 (90.5%) included patients on treatment,^29^, ^30^, ^31^, ^32^, ^33^, ^36^, ^37^, ^38^, ^39^, ^40^, ^41^, ^42^, ^43^, ^44^, ^45^, ^46^, ^47^, ^48^, ^49^ while 2 (9.5%) studies included patients off treatment.^34^, ^35^ Studies recruited individuals with breast (*N* = 8),^32^, ^33^, ^34^, ^35^, ^36^, ^37^, ^41^, ^46^ prostate (*N* = 5),^29^, ^30^, ^31^, ^41^, ^46^ colorectum (*N* = 4),^39^, ^41^, ^43^, ^45^ liquid cancer species (*N* = 4),^38^, ^40^, ^42^, ^44^ pancreatic (*N* = 2),^43^, ^47^ and esophageal (*N* = 1) cancer.^48^ One of these studies included gastrointestinal cancer patients, also recruiting gastric and biliary tract cancer patients.^43^ Cheville et al. also included lung cancer patients (together with those diagnosed with colorectal),^39^ while the study from Zhao et al.^49^ only included head and neck cancer patients.

**TABLE 1:** Studies and participants' characteristics.

Author, year	Sample size	Gender	Group	Group size (*N*)	Retention rate (%)	Mean age (years)	Mean BMI (kg m^−2^)	Treatment phase	Cancer	Cancer stage
An et al.^32^	287	F	STAN HIGH COMB	92 95 100	93.3	49.6 ± 7.9 49.9 ± 8.6 50.4 ± 9.4	25.9 ± 5.0 25.2 ± 4.5 28.1 ± 6.6	On	Breast	I‐IIIA
Bryant et al.^38^	17	M/F	COMB CG	8 9	94.4	52 ± 13 49 ± 15	27.1 ± 3.4 29.6 ± 7.3	On	Leukemia	n/a
Cheville et al.^39^	56	M/F	COMB CG	26 30	84.8	63.8 ± 12.5	n/a	On	Lung, colorectal	IV
Coleman et al.^40^	12	M/F	COMB CG	9 3	–	55 (42–74)^a^ ^,^ ^b^	n/a	On	Multiple myeloma	n/a
Courneya et al.^33^	296	F	STAN HIGH COMB	95 99 102	98.3	49.5 ± 8.0 49.9 ± 8.7 50.4 ± 9.4	26.1 ± 4.9 25.2 ± 4.5 28.3 ± 6.6	On	Breast	I‐IIIc
Demmelmaier et al.^41^	192	M/F	HI‐COMB LMI‐COMB	95 97	66.7	58.1 ± 11.4 59.6 ± 11.8	n/a	On	Breast, prostate, colorectal	n/a
Dieli‐Conwright et al.^34^	91	F	COMB CG	46 45	91	53.5 ± 10.4^b^	n/a	Off	Breast	0–III
Galvao et al.^29^	57	M	COMB CG	29 28	100	69.5 ± 7.3 70.1 ± 7.3	27.4 ± 3.2 28 ± 3.8	On	Prostate	n/a
Hacker et al.^42^	17	M/F	RT CG	8 9	89.5	46.3 ± 16.2^b^	n/a	On	Hematopoietic stem cell transplantation	n/a
Jensen et al.^43^	21	M/F	RT AT	11 10	80.8	58.7 ± 12.0 51.6 ± 13.6	n/a	On	Gastric, colorectal, pancreatic, and biliary tract	n/a
Knols et al.^44^	131	M/F	COMB CG	64 67	100	46.7 ± 13.7 46.6 ± 12.0	22.9 ± 4.3 23.9 ± 4.0	On	Leukemia, lymphoma	n/a
Langlais et al.^30^	21	M	AT RT CG	6 5 10	84	71 (51–84)^a^ ^,^ ^b^	n/a	On	Prostate	Metastatic
Owusu et al.^35^	192	F	COMB CG	94 98	90	71.81 ± 5.99 71.9 ± 5.82	29.8 ± 6.43 30.2 ± 6.90	Off	Breast	I‐III
Piraux et al.^31^	72	M	RT HIIT CG	24 24 24	92	67.9 ± 7.1 67.4 ± 8.9 71.9 ± 8.1	26.1 ± 2.9 26.5 ± 3.9 25.8 ± 4.4	On	Prostate	n/a
Piraux et al.^45^	18	M/F	RT HIIT CG	6 6 6	100	61.5 (52.8; 73.5)^b^ 61.0 (54.5; 65.3)^b^ 64.5 (62.5; 72.5)^b^	27.0 (24.4; 30.1)^b^ 27.0 (25.4; 31.0)^b^ 25.3 (23.3; 28.3)^b^	On	Rectal	II‐III
Schmidt et al.^36^	67	F	RT AT CG	21 20 26	83	53 ± 12.55 56 ± 10.15 54 ± 11.19	n/a	On	Breast	Primary moderate‐ or high‐risk
Sprod et al.^46^	38	M/F	COMB CG	19 19	95	56.6 ± 13.7 63.3 ± 9.4	28.7 ± 5.4 31.3 ± 6.8	On	Prostate, breast	Early‐stage with no metastases/recurrence
Steindorf et al.^37^	160	F	RT CG	80 80	100	55 ± 9.4 56.2 ± 8.6	<25 = 64 25–<30 = 62 30+ = 34	On	Breast	0–III
Steindorf et al.^47^	47	M/F	RT1 RT2 CG	9 21 17	72	62.8 ± 6.4 61 ± 9.3 58.7 ± 8.4	23.5 ± 3.1 22.4 ± 2.9 25.5 ± 5.6	On	Pancreatic	I–IV
van Vulpen et al.^48^	110	M/F	COMB CG	54 56	92	64.3 ± 7.8 63.1 ± 8.5	24.8 ± 3.2 25 ± 3.8	On	Esophageal	I–III
Zhao et al.^49^	18	M/F	COMB CG	11 7	90	57^b^	30 ± 5 32 ± 3	On	Head, neck	n/a

Abbreviations: AT, aerobic training; CG, control group; COMB, combined aerobic and resistance training; HI–COMB, high‐intensity resistance and endurance training; LMI–COMB, low‐to‐moderate intensity resistance and endurance training; Retention rate, the percentage of participants retained at post‐test; RT, resistance training; STAN, standard dose of aerobic exercise, following the Physical Activity Guidelines for Americans endorsed for cancer survivors by the American College of Sports Medicine and the American Cancer Society.

^a^ Data are presented as median and interquartile range.

^b^ Total sample.

**TABLE 2:** Features of the RT interventions.

Author, year	Type	Setting	RT modality	Length (weeks)	Frequency (*n*/week)	Session duration (min)	RT intensity
An et al.^32^	COMB STAN^1^ HIGH^1^	Supervised	Machine‐based (+ aerobic machines)	17 (12–18)	3	50–60	60%–75% 1‐RM (+ AT 55%–75% VO_2_peak)
Bryant et al.^38^	COMB	Supervised	Resistance bands (+ walking/stationary bike)	4	8	15–35	Lighter to heavier 10‐RM (+AT 50%–70% of HRR)
Cheville et al.^39^	COMB	Home‐based	Resistance bands (+ walking)	8	4 or >	90	Incremental (+AT: n/a)
Coleman et al.^40^	COMB	Home‐based	Resistance bands (+ walking/cycling/running)	24	n/a	n/a	RT: n/a (+ AT 9–10 RPE)
Courneya et al.^33^	COMB STAN^1^ HIGH^1^	Mixed	Machine‐based (+ aerobic machines)	17 (12–18)	3	56–60	60%–75% 1‐RM (+AT 55%–75% VO_2_peak)
Demmelmaier et al.^41^	HI‐COMB LMI‐COMB	Mixed	RT: machine‐based, interval training	24	4	n/a	HI: 3 × 6‐RM, 3 × 10‐RM (+AT 80%–90% HRR); LMI: 3 × 12 reps 50% of 6‐RM (+AT 40%–50% of HRR)
Dieli‐Conwright et al.^34^	COMB	Supervised	Machine‐based (+ cardio machines)	16	2–3	80	80%–90% 1‐RM for lower body 60%–70% 1‐RM for upper body (+AT 65%–80% HRmax)
Galvao et al.^29^	COMB	Supervised	Machine‐based (+ cycling and walking/jogging)	12	2	n/a	6–12RM for 2–4 sets/exercise (+AT 65%–80% HRmax)
Hacker et al.^42^	RT	Mixed	Resistance bands	6	3	n/a	Progressive resistance (13 RPE)
Jensen et al.^43^	RT AT^1^	Supervised	Machine‐based	12	2	45	60%–80% 1‐RM
Knols et al.^44^	COMB	Supervised	RT: free weight	12	2	n/a	Progressive resistance (+AT 50%–80% HRmax)
Langlais et al.^30^	RT AT^1^	Home‐based	Machine/free weight	12	3	n/a	n/a
Owusu et al.^35^	COMB	Supervised	Machine/free weight (+ cardio machines)	20	3	60	40%–60% 1‐RM (+AT 50%–70% HRmax)
Piraux et al.^31^	RT HIIT^1^	Supervised	Machine‐based, free weight, bodyweight	5–8	3	30	4–6 RPE
Piraux et al.^45^	RT HIIT^1^	Supervised	Machine‐based, free weight, bodyweight	5	3	30	4–6 RPE
Schmidt et al.^36^	RT AT^1^	Supervised	Machine‐based	12	2	60	50% h1RM
Sprod et al.^46^	COMB	Home‐based	Resistance bands (+ walking)	4	7	n/a	Moderate intensity
Steindorf et al.^37^	RT	Supervised	Machine‐based	12	2	60	60%–80% 1‐RM
Steindorf et al.^47^	RT1 RT2	RT1: supervised RT2: home‐based	RT1: machine‐based RT2: resistance bands	24	2	60	60%–80% 1‐RM
van Vulpen et al.^48^	COMB	Supervised	Free weight (+ rowing ergometer)	12	2	60	15–25 reps at 15–20‐RM (+AT 40‐75%HRR)
Zhao et al.^49^	COMB	Mixed	Free weight (+ walking)	14	3	60	n/a

Abbreviations: 1‐RM, one‐repetition maximum; AT, aerobic training; CG, control group; COMB, combined aerobic and resistance training; h1RM, hypothetical 1 RM; HI–COMB, high‐intensity resistance and endurance training; HIGH, experimental group that follow double the STAN protocol; HIIT, high‐intensity interval training; HRmax, maximal heart rate; HRR, heart rate reserve; LMI–COMB, Low‐to‐moderate intensity resistance and endurance training; Mixed, interventions to some point supervised; RPE, rate of perceived exertion (based on Borg Scale); RT, resistance training; RT1, supervised resistance training; RT2, home‐based training; STAN, standard dose of aerobic exercise, following the Physical Activity Guidelines for Americans endorsed for cancer survivors by the American College of Sports Medicine and the American Cancer Society; VO_2_peak, max peak of oxygen consumption/maximal oxygen consumption.

In general, participants were 57.55 [51.7, 63.7] (Median [IQR]) years of age, with a BMI of 26.5 [25.2, 28.3] kg*m^−2^. The median value of patients' retention rate across studies was 91.5% [84.6, 95.8].

Training modalities performed by the intervention groups in the selected studies consisted of RT alone (*N* = 8; 38.1%),^30^, ^31^, ^36^, ^37^, ^42^, ^43^, ^45^, ^47^ or a combination of RT and AT (*N* = 13; 61.9%).^29^, ^32^, ^33^, ^34^, ^35^, ^38^, ^39^, ^40^, ^41^, ^44^, ^46^, ^48^, ^49^ RT interventions mostly consisted of machine‐based exercises (*N* = 13).^29^, ^30^, ^31^, ^32^, ^33^, ^34^, ^35^, ^36^, ^37^, ^41^, ^43^, ^45^, ^47^ Seven studies applied free weight strategies,^30^, ^31^, ^35^, ^44^, ^45^, ^48^, ^49^ and six studies implemented RT through the use of resistance bands.^38^, ^39^, ^40^, ^42^, ^46^, ^47^ Two of the abovementioned studies also delivered bodyweight training.^31^, ^45^

The majority of the studies had supervised components, with 13^29^, ^31^, ^32^, ^34^, ^35^, ^36^, ^37^, ^38^, ^43^, ^44^, ^45^, ^47^, ^48^ implementing a fully supervised approach and four administering mixed approaches (supervised and home‐based together).^33^, ^41^, ^42^, ^49^ The remaining five studies delivered home‐based interventions.^30^, ^39^, ^40^, ^46^, ^47^ It should be noted that Steindorf et al. presented two RT interventions: one fully supervised and one home‐based.^47^

The median duration of the interventions was 12 [8, 17] weeks, with the majority of the studies with a duration ≥12 weeks (*N* = 15; 71%)^29^, ^30^, ^32^, ^33^, ^34^, ^35^, ^36^, ^37^, ^40^, ^41^, ^43^, ^44^, ^47^, ^48^, ^49^ and with 6 studies (29%) with an intervention shorter than 12 weeks.^31^, ^38^, ^39^, ^42^, ^45^, ^46^ The median frequency of the interventions was 3 [2, 3] times a week, with 9 (43%) studies reporting a frequency of 3 times a week,^30^, ^31^, ^32^, ^33^, ^34^, ^35^, ^42^, ^45^, ^49^ and 7 (33.3%) studies reporting a 2 times a week frequency.^29^, ^36^, ^37^, ^43^, ^44^, ^47^, ^48^ Four more studies presented more than four trainings a week (19%)^38^, ^39^, ^41^, ^46^ and one did not report this information (4.7%).^40^ The median training session duration was 60 [47.5, 60] min. The median value of RT intensity, defined as percentages of the 1RM, was 69% [63.1, 70].

In summary, studies mainly reported a 12‐week supervised RT intervention using free weight and machines, performed 2–3 times a week for 60 minutes per session, with exercise intensity ranging from 60% to 80% of 1RM.

## 4 RISK OF BIAS

**FIGURE 2:**
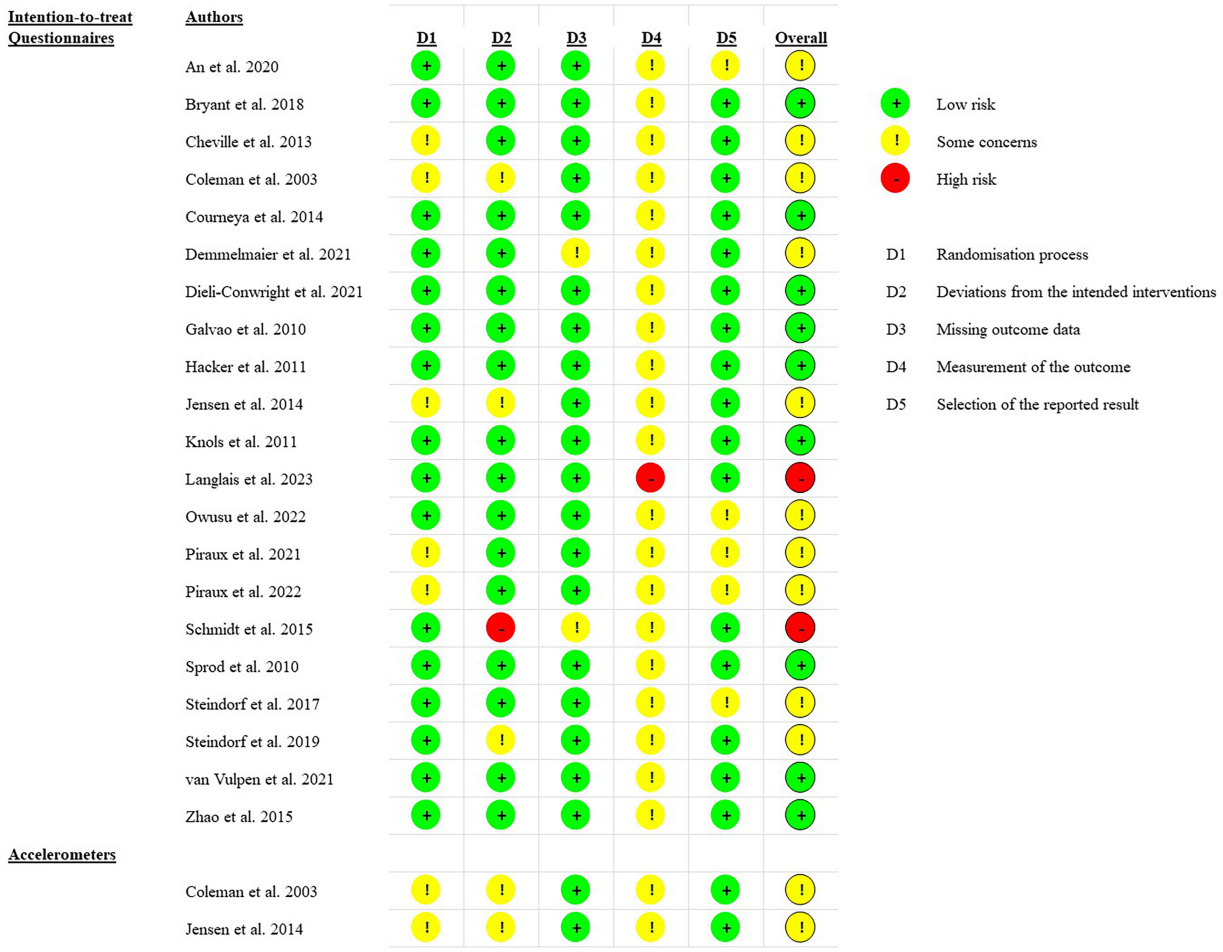
Risk of bias assessment.

RoB evaluations are reported in Figure 2. Across studies, the most frequent overall judgment was “some concerns” (12 out of 23 assessments). When considering domains, the judgment “some concerns” was consistently present in the fourth domain “measurement of the outcome” due to the impossibility to blind the assessors during self‐reported questionnaires. Although a better judgment on this domain should be expected for objective evaluations such as accelerometer sleep assessments, “some concerns” were also found for the two assessments carried out to evaluate these outcomes since this instrument has been shown to be valid for assessing sleep in healthy individuals only.^40^ In general, a medium quality of the studies has been reported, with only two RoB assessments classified as “high RoB”^30^, ^36^ out of 23 judgments.

### 4.1 Study outcomes

Results extracted from 19 studies are reported in Table 3. Two of the included studies did not provide eligible data to be extracted: Cheville et al.^39^ only reported mean difference for sleep outcomes. Hence, it was not possible to calculate the percentage difference from the baseline. Coleman et al. did not report separate data for the intervention and control groups. Instead, results were stratified according to therapeutic strategies, making it impossible to calculate the percentage difference.^40^

**TABLE 3:** Studies results pre–post intervention and percentage differences within groups.

Author, year	Type of RT intervention	Pre‐value, SD	Post‐value SD	%Diff IG	%Diff CG
*Sleep quality*					
*PSQI* ↓					
An et al.^32^	COMB	6.2 ± 4.1	5.2 ± 0.4	−16.1	/
Courneya et al.^33^	COMB	6.2 ± 4.07	6.9 ± 0.30	11.3	/
Dieli‐Conwright et al.^34^	COMB	14.2 ± 3.98	9.4 ± 3.21	−33.9	3.2
Langlais et al.^30^	RT	9 ± 2.8	8 ± 1.6	−11.1	−2.0
Piraux et al.^31^	RT	4 (3.0; 7.5)^t^	4.5 (3.0; 8.5)^t^	12.5	−15.4
Piraux et al.^45^	RT	6.5 (4.0; 9.0) ^t^	6.5 (4.5; 9.0) ^t^	0	41.7
Sprod et al.^46^	COMB	7.06 ± 4.26	6 ± 3.87	−15.0	−4.5
*MOS‐sleep 6* ↓					
Zhao et al.^49^	COMB	34 ± 19	20 ± 16.58	−41.2	−2.4
Total	RT			**0.3**	**8.1**
	COMB			**−19**	**−1.2**
*Sleep disturbance/insomnia*					
PROMIS ↓					
Bryant et al.^38^	COMB	56.1	49.5	−11.9	−2.4
PSQI ↓					
Dieli‐Conwright et al.^34^	COMB	2.3 ± 0.71	1.5 ± 0.38	−34.8	2.4
Owusu et al.^35^	COMB	6.1 ± 3.65	6.1 ± 3.25	−0.7	1.3
Sprod et al.^46^	COMB	1.61 ± 0.78	1.32 ± 0.58	−18.0	2
EORTC QLQ‐C30 ↓					
Galvao et al.^29^	COMB	19.9 ± 27.2	18.6 ± 23.7	−6.5	−2.5
Hacker et al.^42^	RT	29.2 ± 33.0	25 ± 23.6	−14.4	−16.7
Jensen et al.^43^	RT	51.5 ± 40.5	48.5 ± 45.6	−5.8	/
Knols et al.^44^	COMB	30.2 ± 30.1	23 ± 27.4	−23.8	−8.2
Schmidt et al.^36^	RT	36.8 ± 36.67	29.8 ± 31.22	−19.1	12.5
Steindorf et al.^47^	RT1	40.7 ± 27.8	22.2 ± 23.6	−45.5	11.0
Steindorf et al.^47^	RT2	39.7 ± 25.0	33.3 ± 25.8	−16.1	/
Steindorf et al.^47^	RT3 (RT1 + RT2)	40 ± 25.4	30 ± 25.3	−25.0	/
van Vulpen et al.^48^	COMB	25.7 ± 32.81	19.14	−25.5	15.9
ISI ↓					
Piraux et al.^31^	RT	5.5 (2.8;8.5)^t^	6 (3.3; 9.5)^t^	9.1	0
Piraux et al.^45^	RT	8.5 (3.0; 12.8)^t^	9.5 (6.0; 12.8)^t^	11.8	21
Sleep disturbance/insomnia total	RT			**−11.4**	**5.6**
	COMB			**−17.3**	**1.9**
*Daytime sleepiness*					
ESS ↓					
Piraux et al.^31^	RT	5 (3.3; 7.0)^t^	5 (3.0; 10.8)^t^	0	25
Piraux et al.^45^	RT	5.5 (3.5;7.5)^t^	6 (2.8; 9.0)^t^	9.1	−25
Total	RT			**4.5**	**0**
*Sleep, h/d*					
SenseWear ↑					
Demmelmaier et al.^41^	COMB (HI)	7.3 ± 0.9	7.3 ± 1.1	0	/
Demmelmaier et al.^41^	COMB (LMI)	7.2 ± 1.2	7.1 ± 1.3	−1.4	/
*Hours slept during the night* **↑**					
Steindorf et al.^37^	RT	6.6 ± 1.2	6.6 ± 1.3	0	3.1
*Number of awakenings during the night* **↓**					
Steindorf et al.^37^	RT	2 ± 1.3	2.1 ± 1.3	5	0

*Note*: Data are presented as mean ± SD and ^t^median [Q1–Q3]. %Diff percentage differences within the group; bold indicates percentage difference mean values for specific exercise intervention typology. + increase, − decrease, ↑ represent improvements through increase, ↓ represent improvements through decrease.

Abbreviations: AT, aerobic training; CG, control group; COMB, combined aerobic and resistance exercise program; EORTC QLQ‐C30, European Organization for Research and Treatment of Cancer, Quality of life Questionnaire; ESS, Epworth Sleepiness Scale; IG, intervention group; ISI, Insomnia Severity Index; MOS‐Sleep 6, The 6‐item MOS Sleep Problem Index; SenseWear Armband mini (BodyMedia Inc, Pittsburgh, PA, USA); Post‐value, evaluation values after the intervention is completed; Pre‐value, evaluation values before the intervention; PROMIS, Patient‐Reported Outcomes Measurement Information System; PSQI, Pittsburg Sleep Quality Index; RT, resistance training; SD, standard deviation; Type, type of exercise intervention.

Sleeping outcomes were assessed using subjective and objective sleep measures. Outcomes assessed using subjective measurements were sleep quality, sleep disturbance/insomnia, daytime sleepiness, hours slept during the night, and number of awakenings during the night. For the vast majority of data extracted using subjective measures, lower values indicate better sleep, except in the case of hours slept during the night. Outcomes measured were assessed using diverse tools. Sleep quality was calculated using the Pittsburgh Sleep Quality Index (PSQI) in seven studies,^30^, ^31^, ^32^, ^33^, ^34^, ^45^, ^46^ and in one study using the Medical Outcomes Study Sleep Problem Index (MOS‐Sleep 6).^49^ Furthermore, Cheville et al.^39^ rated sleep quality using an 11‐point scale. Sleep disturbance/insomnia was evaluated using four different assessment tools, one study used the Patient‐Reported Outcomes Measurement Information System (PROMIS),^38^ two studies used the Insomnia Severity Index (ISI),^31^, ^45^ while three studies used the PSQI,^34^, ^35^, ^46^ and seven studies extracted data from European Organization for Research and Treatment of Cancer, Quality of life Questionnaire (EORTC QLQ‐C30).^29^, ^36^, ^42^, ^43^, ^44^, ^47^, ^48^ Two studies used the Epworth Sleepiness Scale (ESS) to evaluate daytime sleepiness.^31^, ^45^ Hours slept during the night and number of awakenings during the night were self‐reported by the patients.^37^

Objective sleep measures were assessed solely in two studies with Actigraph (Ambulatory Monitoring, Ardsley, NY) used for determining nighttime sleep minutes and numbers of wake episodes,^40^ and with SenseWear Armband mini (BodyMedia Inc, Pittsburgh, PA, USA) used for monitoring hours a sleep in a day.^41^ Coleman et al. reported a stratification strategy (according to the use of specific drugs) that did not allow the extraction of the results although they reported promising sleep improvements.^40^ Two groups for exercise type were designed to stratify the results: (1) combined aerobic and resistance training (COMB) and (2) resistance training (RT).

### 4.2 Resistance training

Effects of RT on sleep quality were reported by three studies, showing contradictory results.^30^, ^31^, ^45^ One study reported a beneficial effect of RT (−11.1%),^30^ one showed no difference in the intervention group (while the control group showed a worsened sleep quality),^45^ while the third study found a negative influence on sleep quality (12.5%).^31^ The mean percentage difference between studies although demonstrated that RT may avoid worsening of sleep quality (+0.3%) when compared to CGs (+8.1).

The positive impact of RT was reported in insomnia/sleep disturbances when taking into account the mean percentage difference of all studies (−11.4%),^31^, ^36^, ^42^, ^43^, ^45^, ^47^ with only two studies out of six reporting a negative influence.^31^, ^45^ Additionally, resistance training had no effect^31^ or negative impact^45^ on daytime sleepiness. Furthermore, RT training did not affect sleep duration at night, while it negatively impacted the number of awakenings during the night, giving the results of one study (5%).^37^ Results are summarized in Table 3.

### 4.3 Combined aerobic and resistance training

Four studies reported positive effects of COMB training on sleep quality,^32^, ^34^, ^46^, ^49^ while one study reported opposite effects.^33^ Considering the mean percentage difference of all studies combined, the impact of COMB training is shown to have a positive influence (−19%) on sleep quality. Moreover, Cheville et al.^39^ study, which was not included in Table 3 results, reported positive significant improvements sleep quality after a COMB intervention.

The positive effect of COMB training is evident in sleep disturbance/insomnia as well, if we take into account the mean percentage difference of all studies (−17.3%).^29^, ^34^, ^35^, ^38^, ^44^, ^46^, ^48^ Additionally, according to Demmelmaier et al.^41^ study, who reported data tracked by SenseWear, high‐intensity COMB training did not affect the duration of sleep at night (0), while low‐to‐moderate intensity COMB training had an effect on increasing the number of hours of sleep per day (−1.4%).

## 5 META‐ANALYTIC RESULTS

**FIGURE 3:**
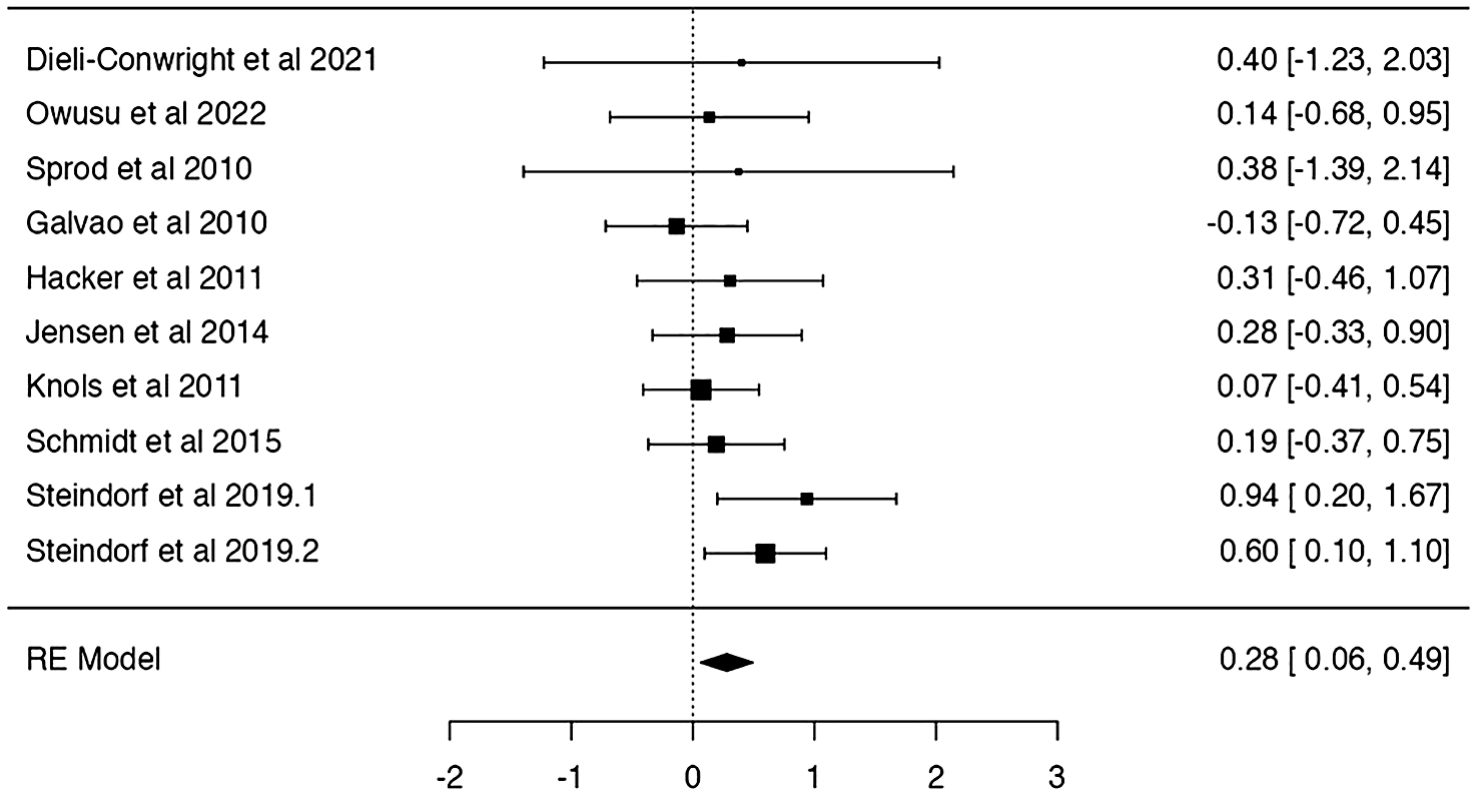
Meta‐analysis results on sleep disturbance/insomnia. Sleep questionnaires use lower scores to indicate symptom reduction. Data signs have been changed, and positive results indicate improvements.

Concerning insomnia and sleep disturbance, the meta‐analytic synthesis supports the positive effects of insomnia reduction between pre‐ and post‐treatment conditions, with an effect of *d* = 0.28 (SE: 0.11, *Z* = 2.51, *p* < 0.01, 95% CI: 0.07–0.49), meaning that participants in the post‐exercise condition reduced their sleep disturbance score compared to the pre‐exercise condition (Figure 3). The *Q*‐test for heterogeneity was non‐significant, meaning that the effect sizes were quite homogeneous (*Q*(9) = 7.56, *p* = 0.58).

**FIGURE 4:**
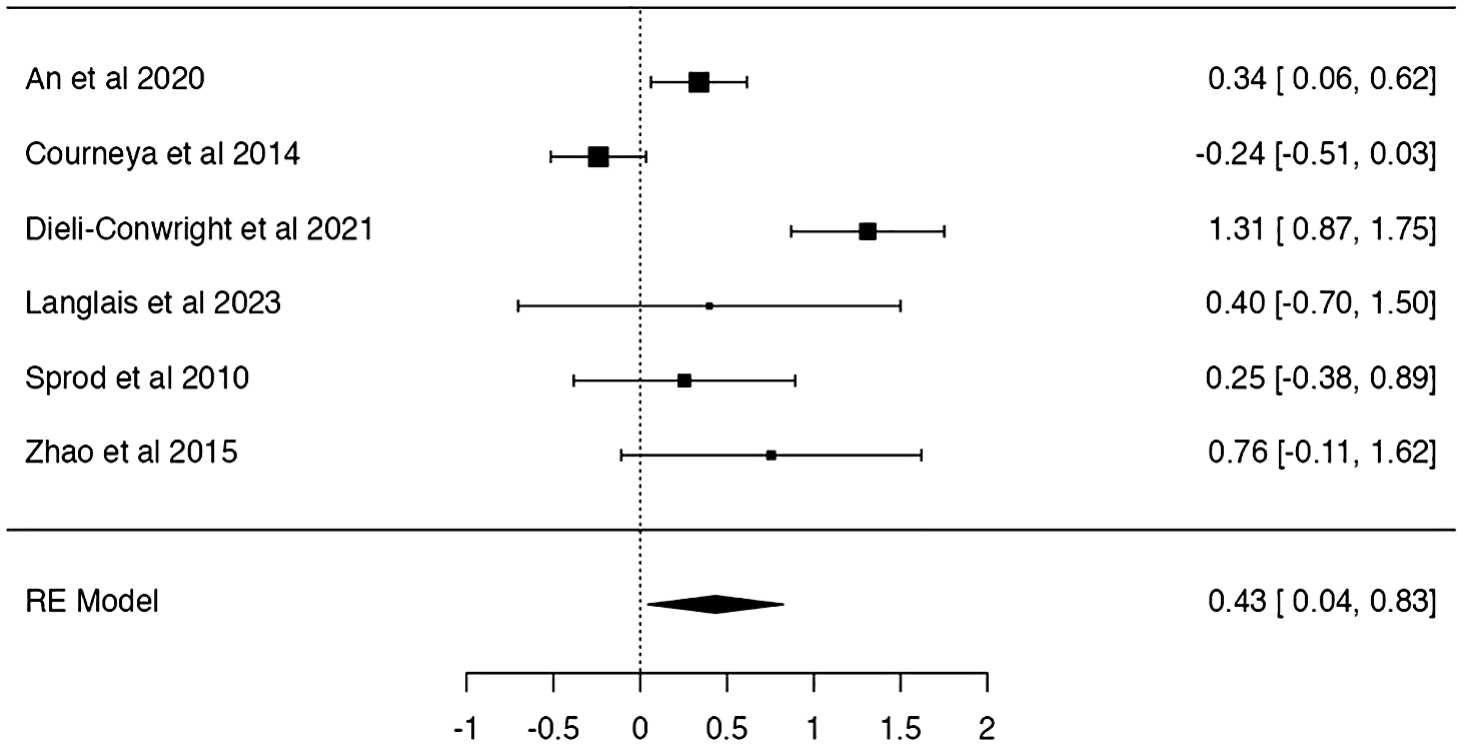
Meta‐analysis results on sleep quality. Sleep questionnaires use lower scores to indicate symptom reduction. Data signs have been changed, and positive results indicate improvements.

Regarding sleep quality, the reported Cohen's *d* was *d* = 0.43 (SE: 0.20, *Z* = 2.18, *p* = 0.029, 95% CI: 0.07–0.49), indicating an improvement of sleep quality after the experimental condition (Figure 4). The *Q*‐test for heterogeneity was significant indicating that the effects are variable (*Q*(5) = 36.20, *p* < 0.001, *I* ^2^ = 75.21%). Due to the small sample size of effects, moderator analysis was not performed.

## 6 DISCUSSION

According to the 21 studies included in this systematic review, we report that RT and COMB training interventions have a somewhat beneficial influcence on sleep quality and sleep disturbance in vast majority of the studies. Furthermore, the meta‐analytic syntheses confirmed the extracted results' consistency, showing a small improvement on sleep outcomes (quality and disturbances) in exercise groups compared to the control.

To the best of our knowledge, this is the first systematic review and meta‐analysis to examine the effect of RT or COMB training on sleep outcomes in patients and survivors suffering from various form of cancers. Three systematic reviews have previously examined the effects of exercise interventions on sleep outcomes in cancer patients.^9^, ^50^, ^51^ However, one review only investigated the effects of walking,^50^ and another^9^ included non‐randomized controlled trials^52^ and studies with only aerobic interventions.^53^, ^54^ Moreover, the systematic review conducted by McGrorry et al.^51^ evaluated the combined intervention of aerobic and resistance training. However, only four studies were included^55^, ^56^, ^57^, ^58^ and the population is limited to female breast cancer survivors. The reviews reported different results, in which one reported no significant effects on sleep outcomes,^9^ while the other two reported improvements for the aerobic and walking exercises on sleep disturbances.^50^, ^51^

Taking into account the results of our systematic review and meta‐analysis beneficial effects of COMB and RT on sleep outcomes are undeniable, however, some inconsistencies may indicate that methodological differences are defining the outcomes of the training interventions and should be explored, and explained in more detail. Our results showed that RT interventions alone limit sleep quality decrease when compared to CGs. This is in contrast with a previous systematic review in which RT has shown to be able to improve sleep quality.^17^ However, this review included a variety of populations (healthy or not) and did not include cancer survivors. Therefore, individuals diagnosed with cancer may present sleep quality reductions which might be dampened by RT. Additionally, only one study of this review assessed sleep quality using the PSQI.^17^ Our results regarding the effects of RT on sleep quality should be interpreted with caution since it was assessed with the PSQI questionnaire by only three studies, showing contrasting results. Langlais et al.^30^ reported a positive change while the other two studies,^31^, ^45^ conducted by the same author, showed opposite effects. However, it is important to note that CGs presented higher sleep quality decreases compared to RT groups. Therefore, even if future studies are needed, the implementation of RT interventions can be a valuable strategy to prevent a decline in sleep quality in cancer populations.

When considering COMB trainings, only one study^33^ of five reports negative effects of COMB training on sleep quality, while the others reported improvements.^32^, ^34^, ^46^, ^49^ Both An et al.^32^ and Courneya et al.^33^ included participants with breast cancer on chemotherapy, reporting different results. However, in Courney et al. study,^33^ sleep quality was assessed 3–4 weeks after chemotherapy. In An et al.^32^ study, the intervention lasted 3–4 weeks after chemotherapy and sleep quality was assessed 6 months after the intervention. These studies assessed sleep quality in different periods after chemotherapy. It is plausible to assume that side effects of chemotherapy were more present in Courneya et al.^33^ than in An et al.^32^ study, which can explain the different outcomes.

When it comes to insomnia/sleep disturbances, positive impact of RT alone was reported (by four studies out of six). In the case of COMB training, all the included studies reported a positive effect on sleep disturbance/insomnia. Although the reason for the positive impact of RT on sleep disturbance is not mentioned by the included studies, we can assume that the links are similar to those in studies that monitor the influence of other training modalities on sleeping outcomes. However, the mechanisms behind the effects of exercise on sleep outcomes are not completely clear. A meta‐analysis indicates that exercise could effectively control inflammation, commonly associated with sleep disorders.^59^ Yet, different mechanisms might be responsible of sleep improvements when exercise is prescribed. These include the thermogenic hypothesis, changes in immune and homeostatic processes, body restoration energy and conservation, increased light exposure, and improved mood.^9^ One of the plausible explanations is the fact that with increased muscle strength, patients can maximize their engagement in physical activity, consequently increasing the homeostatic sleep drive and nighttime sleep.^40^ Additionally, exercise has an effect on reducing anxiety, which is commonly connected to poor sleep.^60^ Thus, we may assume that exercise influenced improvements in anxiety can result in sleep improvement.

A study done by Piraux et al.^31^ reported negative influence of RT on sleep disturbance. Conversely, beneficial effects on cancer‐treatment‐related fatigue, which was the primary outcome, were determined. A plausible explanation for these results, as suggested by authors, is the fact that prostate cancer patients' sleep was disrupted by their increased nocturia due to radiotherapy induced urethra inflammation, making them wake up more often during the night. Additionally, the majority of the participants were undergoing androgen deprivation therapy (ADT) which is known for causing hot flashes directly influencing sleep. It is possible that RT‐positive sleep effects were limited and replaced by negative radiations and ADT symptoms.^31^ Another study by Piraux et al.^45^ points to the negative effect of RT on sleep disturbance. However, the purpose of this study was to examine training interventions feasibility only.^45^

The studies implementing COMB interventions reported positive effects on sleep outcomes. A study by Dieli‐Conwright et al.^34^ suggested that integrating training intervention in the early survivorship phase might generate better sleep outcomes. The authors also suggested that a floor effect may take place with those distant from therapies administration. Nevertheless, the supervised setting in which the intervention was implemented usually elicit greater outcomes and benefits making it safe and valuable.^34^ Another include study by Sprod et al.^46^ hypothesized that the hypothalamic–pituitary–adrenal axis, altered by abnormal cortisol secretion due to radiotherapy, might mediate the connection between the sleep mediators and sleep quality.^46^ The authors suggested that exercise could restore the hormonal axis stimulating cortisol production through the regulation of pro‐inflammatory cytokines, consequently improving sleep outcomes.^46^

Objective sleep measures were assessed solely in two studies with Actigraph (Ambulatory Monitoring, Ardsley, NY) used for determining nighttime sleep minutes and numbers of wake episodes,^40^ and with SenseWear Armband mini (BodyMedia Inc, Pittsburgh, PA, USA) used for monitoring hours a sleep in a day.^41^ While the stratification strategy (according to the use of specific drugs) did not allow us the extraction of Actigraph results from Coleman et al.,^40^ they reported promising sleep improvements mediated by COMB training. On the other hand, studies using SenseWear executed by Demmelmaier et al.^41^ found no effects of high‐intensity COMB training on the duration of sleep at night, while in the case of low‐to‐moderate intensity COMB training led to the increased number of hours of sleep per day. According to studies, Actigraph is used as a worthy substitute for polysomnography, which is the standard for sleep assessments but is not easy to implement being also expensive.^59^ Implementation of objective sleep assessment tools is lacking, even though the importance of implementing both subjective and objective tools for detecting sleep problems is emphasized. A review by Chen et al.^61^ highlights the fact that different methods to measure sleep, especially in those with cancer, can identify diverse sleep problems in the same individual. This is additionally supported by a meta‐analysis reporting disagreements in the results of objective end subjective sleep measures.^59^

Some study limitations should be indicated for this review. Firstly, only two studies included cancer survivors off treatment, making it harder to generalize results for this population. Secondly, only a few studies have investigated the isolated effects of RT although the majority supporting the value and safety of this type of exercise among sleep outcomes. Additionally, only two studies examined sleep outcomes using objective measures, making our conclusions predominantly from questionnaires. Regarding home‐based sessions, it is difficult to establish the intensity since the modality involves the progressive use of resistance bands and the absence of a trainer. Lastly, due to the limited number of study investigating sleep quality and insomnia, moderation analysis was not performed in the meta‐analysis. However, with this review we were able to include a high number of studies (21) with good methodological strategies (RCTs) and only two studies reporting a high RoB judgment.

## 7 CONCLUSIONS

Our results showed the supportive role that both resistance and combined interventions have on sleep outcomes. Given the promising results and the lack of negative evidences of resistance training on sleep outcomes, we may assume that exercise including resistance components is safe for cancer survivors. Still, it is difficult to facilitate exercise prescription having 21 studies with significant methodological differences. Further studies are needed to better define the type of training, duration, and intensity which can provide better sleep improvements, as well as to which cancer population it should be prescribed. Nevertheless, standing on what studies predominantly reported, future resistance training protocols should be administered in supervised settings for 60 minutes, carried out 2–3 times a week for 12 weeks, and with exercise intensity ranging from 60% to 80% of one‐repetition maximum. Additionally, although the effect of resistance or combined aerobic and resistance training positively affects sleep outcomes, there is a lack of objective measures that could support these findings. Future studies, prescribing resistance training only, should combine subjective and objective measurement tools while focusing on sleep as the primary outcome.

## AUTHOR CONTRIBUTIONS

**Dora Maric:** Conceptualization (equal); formal analysis (equal); investigation (equal); writing – original draft (equal); writing – review and editing (equal). **Salvatore Ficarra:** Conceptualization (equal); formal analysis (equal); investigation (equal); methodology (equal); writing – original draft (equal); writing – review and editing (equal). **Luca Di Bartolo:** Conceptualization (equal); formal analysis (equal); investigation (equal); writing – original draft (equal); writing – review and editing (equal). **Carlo Rossi:** Writing – review and editing (equal). **Zoi Asimakopoulou:** Visualization (equal). **Apostolos Vantarakis:** Validation (equal). **Ana Carbonell‐Baeza:** Writing – review and editing (equal). **David Jiménez‐Pavón:** Writing – review and editing (equal). **Beatriz Gomes:** Writing – review and editing (equal). **Paula Tavares:** Visualization (equal). **Rebecca Baxter:** Writing – review and editing (equal). **Susanna Pusa:** Writing – review and editing (equal). **Petra Thaller:** Visualization (equal). **Sofia Papakonstantinou:** Visualization (equal). **Musa Kirkar:** Visualization (equal). **Francesca Glorioso:** Visualization (equal). **Marina Galioto:** Visualization (equal). **Ambra Gentile:** Formal analysis (equal); validation (equal). **Ewan Thomas:** Validation (equal). **Antonino Bianco:** Methodology (equal); supervision (equal).

## FUNDING INFORMATION

The current article is funded by European Commission Project—EU4HEALTH “Outdoor Against Cancer Connects Us”—Project number 101056984.

## CONFLICT OF INTEREST STATEMENT

The authors declare that they have no competing interests.

## Supporting information


**Data S1:**.

## Data Availability

The datasets used and/or analyzed during the current study are available from the corresponding author on reasonable request.
