# Supplementary material for: Effects of resistance training on sleep quality and disorders among individuals diagnosed with cancer: A systematic review and meta‐analysis of randomized controlled trials
Source: Cancer Med. 2024 Apr 23;13(8):e7179. doi: 10.1002/cam4.7179 (PMC11036080; doi:10.1002/cam4.7179)
Supplement: Supplementary file 1 — Data S1:. [file CAM4-13-e7179-s001.docx]

The Supplementary file presents:

1. Search strategy

3. Search strategy and results for each database included

3. Title, Abstract and Full-text screening guide

4. Level of evidence and grades of recommendations

| **1. Search strategy** |
| --- |
| **Population** |
| patients diagnosed with cancer all stages/types, before, during and after treatment. |
| **Interventions** |
| Exercise interventions with resistance training component (with or without aerobic component). |
| **Comparators** |
| Usual care, wait-list control, stretching, non-exercising |
| **Outcomes** |
| Objective sleep-related outcomes: self-report questionnaires, polysomnography, time-lapse photography, television camera and video footage. |
| **Study design** |
| Randomized Controlled Trials |
| **Limitations** |
| Peer-review papers written in English or Italian |
| **Date of Publication** |
| No limit to the Date of publication was applied |

| **2. Search strategy and results for each database included** |
| --- |

| Database | Search | #Retrieved |
| --- | --- | --- |
| PubMed/Medline (NLM) | | |
| #1 | (exercise[MeSH Terms]) OR (exercise) OR (exercises) OR (resistance exercise[MeSH Terms]) OR (resistance exercise) OR (resistance exercises) OR (resistance training[MeSH Terms]) OR (resistance training) OR (resistance trainings) OR (anaerobic exercise[MeSH Terms]) OR (anaerobic exercise) OR (anaerobic exercises) OR (anaerobic training[MeSH Terms]) OR (anaerobic training) OR (anaerobic trainings) OR (aerobic exercise[MeSH Terms]) OR (aerobic exercise) OR (aerobic exercises) OR (aerobic training[MeSH Terms]) OR (aerobic training) OR (aerobic trainings) OR (endurance training[MeSH Terms]) OR (endurance training) OR (endurance trainings) OR (circuit-based exercise[MeSH Terms]) OR (circuit-based exercise) OR (circuit-based exercises) OR (exercise therapy[MeSH Terms]) OR (exercise therapy) OR (exercise therapies) OR (exercise training[MeSH Terms]) OR (exercise training) OR (exercise trainings) OR (gymnastic[MeSH Terms]) OR (gymnastic) OR (high-intensity interval training[MeSH Terms]) OR (high-intensity interval training) OR (high-intensity interval trainings) OR (HIIT[MeSH Terms]) OR (HIIT) OR (isometric exercise[MeSH Terms]) OR (isometric exercise) OR (isometric exercises) OR (isometric training[MeSH Terms]) OR (isometric training) OR (isometric trainings) OR (jogging[MeSH Terms]) OR (jogging) OR (nordic walking[MeSH Terms]) OR (nordic walking) OR (physical activity[MeSH Terms]) OR (physical activity) OR (physical activities) OR (physical exercise[MeSH Terms]) OR (physical exercise) OR (physical exercises) OR (plyometric exercise[MeSH Terms]) OR (plyometric exercise) OR (plyometric exercises) OR (plyometric training[MeSH Terms]) OR (plyometric training) OR (plyometric trainings) OR (preoperative exercise[MeSH Terms]) OR (preoperative exercise) OR (preoperative exercises) OR (resistance band) OR (resistance bands) OR (running[MeSH Terms]) OR (running) OR (stair climbing[MeSH Terms]) OR (stair climbing) OR (strength exercise[MeSH Terms]) OR (strength exercise) OR (strength exercises) OR (strength training[MeSH Terms]) OR (strength training) OR (strength trainings) OR (suspension exercise[MeSH Terms]) OR (suspension exercise) OR (suspension exercises) OR (suspension training[MeSH Terms]) OR (suspension training) OR (suspension trainings) OR (swimming[MeSH Terms]) OR (swimming) OR (walking[MeSH Terms]) OR (walking) OR (weight training[MeSH Terms]) OR (weight training) OR (weight trainings) OR (weightlifting) OR (weight lifting[MeSH Terms]) OR (weight lifting) |  |
| #2 | (neoplasm[MeSH Terms]) OR (neoplasm) OR (neoplasms) OR (cancer[MeSH Terms]) OR (cancer) OR (cancers) OR (cancer patient[MeSH Terms]) OR (cancer patient) OR (cancer patients) OR (cancer survivor[MeSH Terms]) OR (cancer survivor) OR (cancer survivors) OR (cancer survivorship[MeSH Terms]) OR (cancer survivorship) OR (chemotherapy[MeSH Terms]) OR (chemotherapy) OR (immunotherapy[MeSH Terms]) OR (immunotherapy) OR (leukemia[MeSH Terms]) OR (leukemia) OR (leukemias) OR (leukaemia[MeSH Terms]) OR (leukaemia) OR (leukaemias) OR (lymphoma[MeSH Terms]) OR (lymphoma) OR (lymphomas) OR (palliative chemotherapy[MeSH Terms]) OR (palliative chemotherapy) OR (radiation therapy[MeSH Terms]) OR (radiation therapy) OR (radiotherapy[MeSH Terms]) OR (radiotherapy) OR (tumor[MeSH Terms]) OR (tumor) OR (tumors) OR (tumour[MeSH Terms]) OR (tumour) OR (tumours) |  |
| #3 | (sleep[MeSH Terms]) OR (sleep) OR (circadian rhythm) OR (circadian rhythms) OR (insomnia[MeSH Terms]) OR (insomnia) OR (irregular sleep[MeSH Terms]) OR (irregular sleep) OR (non-restorative sleep[MeSH Terms]) OR (non-restorative sleep) OR (polysomnography[MeSH Terms]) OR (polysomnography) OR (sleep apnea[MeSH Terms]) OR (sleep apnea) OR (sleep behaviour[MeSH Terms]) OR (sleep behaviour) OR (sleep behaviours) OR (sleep behavior[MeSH Terms]) OR (sleep behavior) OR (sleep behaviors) OR (sleep deprivation[MeSH Terms]) OR (sleep deprivation) OR (sleep diary) OR (sleep diaries) OR (sleep disorder[MeSH Terms]) OR (sleep disorder) OR (sleep disorders) OR (sleep disturbance[MeSH Terms]) OR (sleep disturbance) OR (sleep disturbances) OR (sleep duration[MeSH Terms]) OR (sleep duration) OR (sleep habit[MeSH Terms]) OR (sleep habit) OR (sleep habits) OR (sleep hygiene[MeSH Terms]) OR (sleep hygiene) OR (sleep latency[MeSH Terms]) OR (sleep latency) OR (sleep movement disorder[MeSH Terms]) OR (sleep movement disorder) OR (sleep movement disorders) OR (sleep pattern) OR (sleep patterns) OR (sleep problem[MeSH Terms]) OR (sleep problem) OR (sleep problems) OR (sleep quality[MeSH Terms]) OR (sleep quality) OR (sleep stage[MeSH Terms]) OR (sleep stage) OR (sleep stages) OR (sleepiness[MeSH Terms]) OR (sleepiness) OR (sleeping habit[MeSH Terms]) OR (sleeping habit) OR (sleeping habits) OR (sleeping quality) |  |
| #4 | #1 AND #2 AND #3 | 4.818 results |
| Scopus | | |
| #1 | TITLE-ABS-KEY ( {exercise} OR {resistance exercise} OR {resistance training} OR {anaerobic exercise} OR {anaerobic training} OR {aerobic exercise} OR {aerobic training} OR {endurance training} OR {circuit-based exercise} OR {exercise therapy} OR {exercise training} OR {gymnastic} OR {high-intensity interval training} OR {HIIT} OR {isometric exercise} OR {isometric training} OR {jogging} OR {nordic walking} OR {physical activity} OR {physical exercise} OR {plyometric exercise} OR {plyometric training} OR {preoperative exercise} OR {resistance band} OR {running} OR {stair climbing} OR {strength exercise} OR {strength training} OR {suspension exercise} OR {suspension training} OR {swimming} OR {walking} OR {weight training} OR {weightlifting} OR {weight lifting} ) |  |
| #2 | TITLE-ABS-KEY ( {neoplasm} OR {cancer} OR {cancer patient} OR {cancer survivor} OR {cancer survivorship} OR {chemotherapy} OR {immunotherapy} OR {leukemia} OR {lymphoma} OR {palliative chemotherapy} OR {radiation therapy} OR {radiotherapy} OR {tumor} ) |  |
| #3 | TITLE-ABS-KEY ( {sleep} OR {circadian rhythm} OR {insomnia} OR {irregular sleep} OR {non-restorative sleep} OR {polysomnography} OR {sleep apnea} OR {sleep behaviour} OR {sleep deprivation} OR {sleep diary} OR {sleep disorder} OR {sleep disturbance} OR {sleep duration} OR {sleep habit} OR {sleep hygiene} OR {sleep latency} OR {sleep movement disorder} OR {sleep pattern} OR {sleep problem} OR {sleep quality} OR {sleep stage} OR {sleepiness} OR {sleeping habit} OR {sleeping quality} ) |  |
|  | #1 AND #2 AND #3 | 3.825 |
| Web of Science | | |
| #1 | ALL=(“exercise” OR “resistance exercise” OR “resistance training” OR “anaerobic exercise” OR “anaerobic training” OR “aerobic exercise” OR “aerobic training” OR “endurance training” OR “circuit-based exercise” OR “exercise therapy” OR “exercise training” OR “gymnastic” OR “high-intensity interval training” OR “HIIT” OR “isometric exercise” OR “isometric training” OR “jogging” OR “nordic walking” OR “physical activity” OR “physical exercise” OR “plyometric exercise” OR “plyometric training” OR “preoperative exercise” OR “resistance band” OR “running” OR “stair climbing” OR “strength exercise” OR “strength training” OR “suspension exercise” OR “suspension training” OR “swimming” OR “walking” OR “weight training” OR “weightlifting” OR “weight lifting”) |  |
| #2 | ALL=(“neoplasm” OR “cancer” OR “cancer patient” OR “cancer survivor” OR “cancer survivorship” OR “chemotherapy” OR “immunotherapy” OR “leukemia” OR “leukaemia” OR “lymphoma” OR “palliative chemotherapy” OR “radiation therapy” OR “radiotherapy” OR “tumor” OR “tumour”) |  |
| #3 | ALL=(“sleep” OR “circadian rhythm” OR “insomnia” OR “irregular sleep” OR “non-restorative sleep” OR “polysomnography” OR “sleep apnea” OR “sleep behaviour” OR “sleep behavior” OR “sleep deprivation” OR “sleep diary” OR “sleep disorder” OR “sleep disturbance” OR “sleep duration” OR “sleep habit” OR “sleep hygiene” OR “sleep latency” OR “sleep movement disorder” OR “sleep pattern” OR “sleep problem” OR “sleep quality” OR “sleep stage” OR “sleepiness” OR “sleeping habit” OR “sleeping quality”) |  |
| #4 | #1 AND #2 AND #3 | 2.173 |
| Cochrane Central Register of Controlled Trials (Wiley} | | |
| #1 | MeSH descriptor: [Exercise] explode all trees |  |
| #2 | (“exercise”) OR (exercise NEXT (therapy or training)) OR (resistance NEXT (exercise* or training*)) OR (anaerobic NEXT (exercise* or training*)) OR (aerobic NEXT (exercise* or training*)) OR (“endurance training” OR “circuit-based exercise” OR “gymnastic” OR “high-intensity interval training” OR “HIIT”) OR (isometric NEXT (exercise* or training*)) OR (“jogging” OR “nordic walking”) OR (physical NEXT (activity* or exercise*)) OR (plyometric NEXT (exercise* or training*)) OR (“preoperative exercise” OR “resistance band” OR running OR “stair climbing”) OR (strength NEXT (exercise* or training*)) OR (suspension NEXT (exercise* or training*)) OR (“swimming” OR “walking” OR “weight training” OR “weightlifting” OR “weight lifting”) |  |
| #3 | MeSH descriptor: [Neoplasms] explode all trees |  |
| #4 | (“neoplasm”) OR (cancer NEXT (patient* or survivor* or survivorship)) OR (“chemotherapy” OR “immunotherapy” OR “leukemia” OR “leukaemia” OR “lymphoma” OR “palliative chemotherapy” OR “radiation therapy” OR “radiotherapy” OR “tumor*” or “tumour*”) |  |
| #5 | MeSH descriptor: [Sleep] explode all trees |  |
| #6 | (“sleep”) OR (“circadian rhythm” OR “insomnia” OR “irregular sleep” OR “non-restorative sleep” OR “polysomnography”) OR (sleep NEXT (“apnea” or “behaviour” or “behavior” or “deprivation” or “diary” or “disorder*” or “disturbance” or “duration” or “habit*” or “hygiene” or “latency” or “movement disorder” or “pattern” or “problem*” or “quality*” or “stage”)) OR (“sleepiness”) OR (sleeping NEXT (habit* or quality*)) |  |
| #7 | #1 OR #2 |  |
| #8 | #3 OR #4 |  |
| #9 | #5 OR #6 |  |
| #10 | #7 AND #8 AND #9 | 846 |

**3. Title and Abstract screening - Review Strategies**

Step 1. Title and Abstract screening.

Use the YES, MAYBE, NO button to asses studies eligibility.

- Logic Scheme to assess eligibility:

1. Participants: Are the included participants diagnosed with cancer? If not click “NO” and exclude the record. If yes go to step 2.
2. Intervention: Is the study prescribing exercise intervention containing resistance training? If not click “NO” and exclude it. If yes go to step 3.
3. Design: Is the study a Randomized Controlled Trial? If not click “NO” and exclude it. If yes go to step 4.
4. Outcome: Is the study assessing at least one sleep-related variable? If not click “NO” and exclude it. If yes click “YES” and include the record. If unsure of one or all of the previous questions click “MAYBE” (website version) or click “Skip” on the mobile app.

Step 2. Full-text Screening

Use the YES, NO button to asses studies eligibility.

- Logic Scheme to assess eligibility:

| **Questions** | **Answers** | |
| --- | --- | --- |
| Are the included participants diagnosed with cancer? | If yes go to step 2.  (All cancer types, stages, therapy phases). | If not click “NO” and exclude the record selecting the following reason:  PARTICIPANTS  (Animal studies, healthy participants, other disease other than cancer and impossibility to isolate cancer participants’ data). |
| Is the study prescribing exercise intervention containing resistance training (RT)? | If yes go to step 3.  (RT component is included in the intervention; RT + aerobic training). | If not click “NO” and exclude the record selecting the following reason:  INTERVENTION  (No exercise and/or RT component, RT is part of a multidisciplinary approach, single session study). |
| Is the study a Randomized Controlled Trial (RCT)? | If yes go to step 4.  (RCT or Pilot RCT) | If not click “NO” and exclude the record selecting the following reason:  STUDY DESIGN  (Everything that’s not RCT) |
| Is the study assessing at least one sleep-related variable? | If yes click “YES” and include the record.  (Any standardized sleep-related outcome: e.g. questionnaires, polysomnography, time-lapse photography, television camera and video footage) | If not click “NO” and exclude the record selecting the following reason:  OTUCOME  (Only non-sleep-related outcomes or non standardized test/questionnaire) |

| **4. Levels of evidence and grades of recommendations** | | |
| --- | --- | --- |
| **Author** | **Level of Evidence** | **Grades of Recommendation** |
| An et al 2020 | 1b | A |
| Bryant et al 2018 | 2b | C |
| Cheville et al 2013 | 2b | B |
| Coleman et al 2003 | 2b | C |
| Courneya et al 2014 | 1b | A |
| Demmelmaier et al 2021 | 2b | B |
| Dieli-Conwright et al 2021 | 1b | A |
| Galvao et al 2010 | 2b | B |
| Hacker et al 2011 | 2b | C |
| Jensen et al 2014 | 2b | C |
| Knols et al 2011 | 1b | A |
| Langlais et al 2023 | 2b | C |
| Owusu et al 2022 | 1b | A |
| Piraux et al 2021 | 1b | A |
| Piraux et al 2022 | 2b | B |
| Schmidt et al 2015 | 2b | B |
| Sprod et al 2010 | 2b | B |
| Steindorf et al 2017 | 1b | A |
| Steindorf et al 2019 | 2b | C |
| van Vulpen et al 2021 | 1b | A |
| Zhao et al 2015 | 2b | C |
| Level of Evidence 1A= Systematic review (with homogeneity) of RCTs; 1B=Individual RCT (with narrow confidence intervals); 1C=All or none study; 2A=Systematic review (with homogeneity) of cohort studies; 2B= Individual Cohort study (including low quality RCT, e.g. <80% follow-up); 2C=“Outcomes” research; Ecological studies; 3A= Systematic review (with homogeneity) of case-control studies; 3B=Individual Case-control study; 4= Case series (and poor quality cohort and case-control study; 5=Expert opinion without explicit critical appraisal or based on physiology bench research or “first principles” *From the Centre for Evidence-Based Medicine, <http://www.cebm.net>. Grades of Recommendation A= Level 1, Strong recommendation; B=Levels 2, 3 or 4, Recommendation; C=Levels 2, 3 or 4, Option; D=Level 5, Option. From American Society of Plastic Surgeons Evidence-based clinical practice guidelines. Available at: <https://www.plasticsurgery.org/documents/medical-professionals/health-policy/evidence-practice/ASPS-Scale-for-Grading-Recommendations.pdf>. Accessed February 12, 2021. | | |
